# Familial risk of autism alters subcortical and cerebellar brain anatomy in infants and predicts the emergence of repetitive behaviors in early childhood

**DOI:** 10.1002/aur.2083

**Published:** 2019-02-22

**Authors:** Inês Pote, Siying Wang, Vaheshta Sethna, Anna Blasi, Eileen Daly, Maria Kuklisova‐Murgasova, Sarah Lloyd‐Fox, Evelyne Mercure, Paula Busuulwa, Vladimira Stoencheva, Tony Charman, Steven C. R. Williams, Mark H. Johnson, Declan G. M. Murphy, Grainne M. McAlonan, The BASIS Team

**Affiliations:** ^1^ Sackler Institute for Translational Neurodevelopment, Department of Forensic and Neurodevelopmental Sciences, Institute of Psychiatry, Psychology & Neuroscience King's College London London UK; ^2^ Centre for the Developing Brain, Division of Imaging Sciences and Biomedical Engineering King's College London London UK; ^3^ Institute of Biomedical Engineering, Department of Engineering Science University of Oxford Oxford UK; ^4^ Centre for Brain and Cognitive Development, Birkbeck University of London London UK; ^5^ Institute of Cognitive Neuroscience University College London London UK; ^6^ GKT School of Medical Education King's College London London UK; ^7^ Department of Psychology, Institute of Psychiatry, Psychology & Neuroscience King's College London London UK; ^8^ Department of Neuroimaging, Institute of Psychiatry, Psychology & Neuroscience King's College London London UK; ^9^ NIHR Biomedical Research Centre for Mental Health at the South London and Maudsley NHS Foundation Trust and King's College London London UK

**Keywords:** autism spectrum disorder, infants, familial risk, magnetic resonance imaging—structural, cerebellum, subcortex, mother–infant interaction

## Abstract

Autism spectrum disorder (ASD) is a common neurodevelopmental condition, and infant siblings of children with ASD are at a higher risk of developing autistic traits or an ASD diagnosis, when compared to those with typically developing siblings. Reports of differences in brain anatomy and function in high‐risk infants which predict later autistic behaviors are emerging, but although cerebellar and subcortical brain regions have been frequently implicated in ASD, no high‐risk study has examined these regions. Therefore, in this study, we compared regional MRI volumes across the whole brain in 4–6‐month‐old infants with (high‐risk, *n* = 24) and without (low‐risk, *n* = 26) a sibling with ASD. Within the high‐risk group, we also examined whether any regional differences observed were associated with autistic behaviors at 36 months. We found that high‐risk infants had significantly larger cerebellar and subcortical volumes at 4–6‐months of age, relative to low‐risk infants; and that larger volumes in high‐risk infants were linked to more repetitive behaviors at 36 months. Our preliminary observations require replication in longitudinal studies of larger samples. If correct, they suggest that the early subcortex and cerebellum volumes may be predictive biomarkers for childhood repetitive behaviors. ***Autism Res** 2019, 12: 614–627*. © 2019 The Authors. *Autism Research published by International Society for Autism Research* published byWiley Periodicals, Inc.

**Lay Summary:**

Individuals with a family history of autism spectrum disorder (ASD) are at risk of ASD and related developmental difficulties. This study revealed that 4–6‐month‐old infants at high‐risk of ASD have larger cerebellum and subcortical volumes than low‐risk infants, and that larger volumes in high‐risk infants are associated with more repetitive behaviors in childhood.

**Abbreviations**
ADI‐R: Autism Diagnostic Interview—Revised
ADOS‐2: Autism Diagnostic Observation Schedule—Second Edition
ANCOVA: analysis of covariance
ASD: autism spectrum disorder
DSM‐5: Diagnostic and Statistical Manual of Mental Disorders
GMM: Gaussian mixture model
GRS: global ratings scale
ICC: intra‐class correlation coefficient
MSEL: Mullen scales of early learning
SPM: statistical parametric mapping
T2w: T2‐weighted.

## Introduction

Autism spectrum disorder (ASD) is a common heterogeneous neurodevelopmental condition, characterized by difficulties in reciprocal social communication and social interaction, restricted and repetitive patterns of behavior, and atypical sensory responses (American Psychiatric Association, 2013). Given ASD's genetic liability, infant siblings of diagnosed children are at risk of developing a range of autistic features, with up to 20% reaching the threshold for a clinical diagnosis, and a further 15–20% developing autistic features consistent with the broader autism phenotype (Constantino, Zhang, Frazier, Abbacchi, & Law, 2010; Georgiades et al., 2013; Ozonoff et al., 2011).

There is also accumulating evidence that brain and behavior is altered in high‐risk infants within the first year of life. For example, increased area and thickness of the corpus callosum (Wolff et al., 2015) as well as increased volume of subarachnoid cerebrospinal fluid (CSF) (Shen et al., 2013, 2017), starting at 6 months, is positively correlated with subsequent ASD symptom severity at 24 months (Shen et al., 2013, 2017; Wolff et al., 2015). In addition, high‐risk infants who are later diagnosed with ASD have significantly enlarged brain volumes and an increase in total brain growth between 12 and 24 months, when compared to infants who do not develop the disorder (Hazlett et al., 2017; Shen et al., 2013). Preceding this period of brain overgrowth, between 6 and 12 months of age, high‐risk infants have been reported with hyperexpansion of the cortical surface area and atypical connectivity across functional brain networks, which predict later ASD diagnosis (Emerson et al., 2017; Hazlett et al., 2017). Thus, increasing (albeit preliminary) evidence suggests that infants at high‐risk of ASD have early differences in brain structure and function at 6 months, which are associated with subsequent development of ASD symptoms. However, it is still unclear whether anatomical differences can be identified at younger ages and/or in other brain regions. For example, although cerebellar abnormalities are among the most frequently reported findings in ASD literature (Becker & Stoodley, 2013; Fatemi et al., 2012; Rogers et al., 2013), and subcortical brain regions have been linked to ASD in older cohorts (Estes et al., 2011; Langen et al., 2014; Langen, Durston, Staal, Palmen, & van Engeland, 2007; Stanfield et al., 2008), no study has yet explored whether the development of these regions is altered in very young infants at risk of ASD.

Therefore, in this prospective study, our primary objective was to use MRI to investigate differences in total and regional—including subcortical and cerebellar—brain volumes in 4–6‐month‐old infants at high familial risk of ASD, relative to a group of infants at low‐risk. We next examined whether any differences observed in the high‐risk group were associated with the subsequent emergence of autistic symptoms at 36 months. We anticipated that the majority of high‐risk infants would not go on to receive a diagnosis of ASD; therefore, we adopted a dimensional approach and examined the potential link between regional brain volume in infancy and behavioral outcomes across the high‐risk group in childhood. Finally, as we previously reported that sensitive early care (i.e., warm, accepting, nondemanding, and nonintrusive maternal response to the infant's communication cues) is associated with differences in subcortical and total gray matter volume in typical infants (Sethna et al., 2017), we undertook an exploratory analysis of whether early mother–infant interaction dimensions (maternal sensitivity and remoteness; infant communication; and fretfulness) moderated the extent of any group differences in brain anatomy observed.

## Methods

### *Participants*

Data was collected from 59 infants, of whom nine were excluded from the main analysis: eight due to poor image quality, and one low‐risk participant because of a subsequent ASD diagnosis. Hence, the final sample consisted of 50 infants: 26 low‐risk (*n* = 12 male; mean age = 4.81 months, SD = 0.69) and 24 high‐risk (*n* = 11 male; mean age = 4.79 months, SD = 0.72). Participants were characterized as high‐risk if their parents provided a clear history that their older full sibling had been assessed by the appropriate clinical services and found to have a clinical diagnosis of ASD. This was further confirmed by reference to the SCQ (Social Communication Questionnaire; Rutter, Bailey, & Lord, 2003) and the DAWBA (Development and Well‐being Assessment; Goodman, Ford, Richards, Gatward, & Meltzer, 2000), which requires a clinician opinion in addition to a structured account of the older sibling's behaviors. These are well‐validated standard measures to support a diagnosis of ASD that have been shown to discriminate between ASD and non‐ASD cases with high sensitivity and specificity (Chandler et al., 2007; McEwen et al., 2016). Participants were considered low‐risk if they had no family history of a first‐degree relative with ASD.

Infants in the high‐risk group were recruited *via* the BASIS (British Autism Study of Infant Siblings) network, and those in the low‐risk group were recruited from the local community. Written informed consent was obtained from parents. The study was approved by the BASIS network and received ethics approval from the UK National Research Ethics Service (REC 08/H0718/76 and 06/MRE02/73). Initial exclusion criteria prior to enrolment in the study included: (a) preterm infants (born <36 weeks' gestation), with (b) contraindications for MRI (for example, metallic implants), (c) congenital abnormalities, (d) major complications in pregnancy and/or delivery (such as perinatal asphyxia or seizures), (e) evidence of a genetic condition or syndrome reported to be associated with ASD (as is fragile X syndrome), (f) born to mothers with any current or past major psychiatric illness (such as major mood disorder or schizophrenia), and (g) with poor working knowledge of the English language (precluding informed consent). An in‐house semi‐structured interview was used to review medical and psychiatric history, and the family doctor of each participant was also informed that their patient was participating in the study.

### *Imaging Procedures*

#### *Image acquisition*

At 4–6 months of age, infants were scanned during natural sleep at the Centre for Neuroimaging in the Institute of Psychiatry, Psychology, and Neuroscience at King's College London, using the same 1.5T General Electric Twinspeed scanner (GE Medical Systems, Milwaukee, WI) equipped with an eight‐channel head coil (Blasi et al., 2011). To reduce movement during scanning, infants were swaddled in a sheet and comfortably positioned in a Med‐Vac Infant Immobilization Bag (CFI Medical Solutions). To minimize acoustic noise, the scanner bore was lined with sound attenuating foam (Ultra Barrier, American Micro Industries), and the infant's ears were protected with both MiniMuff noise attenuators (Natus Medical) and MR‐compatible piezoelectric headphones (MR Confon). A pulse oximeter secured onto the infant's toe enabled monitoring of the heart rate and blood oxygen saturation levels. The imaging data available for most infants was from a rapidly acquired T2‐weighted (T2w) fast spin echo protocol: repetition time = 3000/4500 ms, echo time = 15 ms, slice thickness = 4 mm, slice gap = 2 mm, field of view = 180 mm, flip angle = 90**°**, and a 256 × 224 matrix. A radiologist who was unaware of risk group reviewed all scans to exclude obvious incidental abnormalities.

#### *Image processing and segmentation*

**Figure 1:**
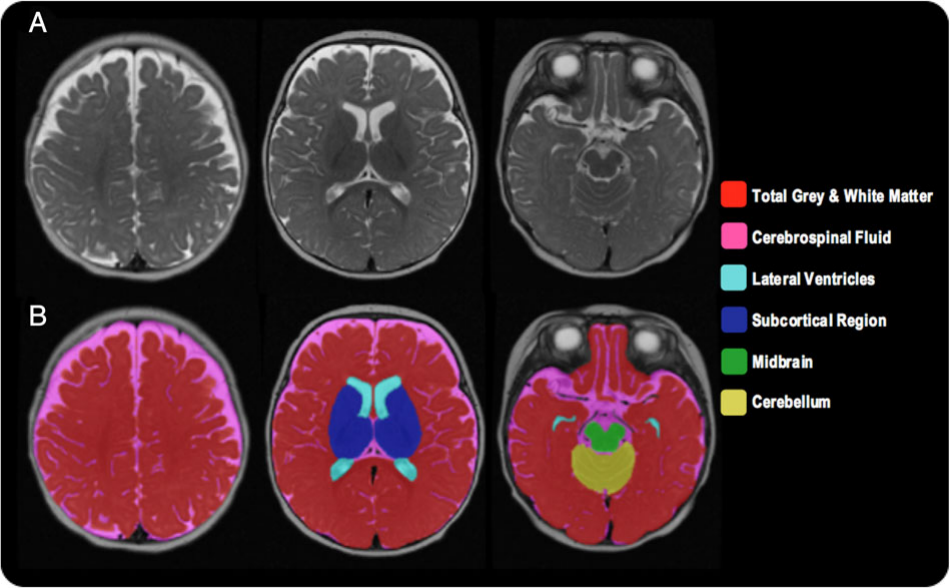
Volumetric segmentation of a 4–6‐month infant brain, where (A) is the axial T2‐weighted acquisition and (B) is the output of the final segmentation.

The T2w images (Fig. 1A) were segmented following an automated protocol for low‐resolution images, developed in‐house (Sethna et al., 2017). In sum, T2w images were skull‐stripped and the masked images segmented using an atlas‐based method, which used a 4D probabilistic neonatal brain atlas (Kuklisova‐Murgasova et al., 2011) as an input to the adapted Statistical Parametric Mapping software (SPM8). Iterated Conditional Modes were then applied to enhance the Gaussian Mixture Model (GMM) parameters for the tissue intensity distributions, the bias field parameters, and the atlas deformation parameters. Following this, the segmented CSF was refined, and the partial volume misclassifications were corrected using second‐order Markov random fields. Any errors from the automated segmentation were manually corrected (blind to infant risk group and mother–infant interaction ratings) using ITK‐SNAP (Yushkevich et al., 2006). The following brain volumes were obtained: total gray plus white matter, CSF (including third and fourth ventricles), lateral ventricles, subcortical region (including caudate nucleus, putamen, globus pallidus, thalamus, and internal capsule), midbrain (including cerebral peduncle, substantia nigra, brainstem, and pons), and cerebellum (Fig. 1B). Due to ongoing myelination, the tissue contrast in 4–6‐month‐old brains does not easily allow for a reliable differentiation of gray and white matter; therefore, similar to others (Hazlett et al., 2012), gray and white matter tissue classes were not further segmented. The cerebellum and subcortical region segmentations thus include both gray and white matter. Total brain matter volume was defined as the sum of all brain regions (excluding CSF and lateral ventricles), whereas intracranial volume was defined as the sum of all regions.

#### *Reliability of volumetric segmentation*

The validity of the automated segmentation protocol was assessed by comparing its output with the output of manually segmented (i.e., “gold standard”) brains, randomly selected from 20% (i.e., *n* = 10) of the individuals (mean age = 4.52months; *n* = 3 male). All of the manual segmentations were completed on ITK‐SNAP (i.e., a software application used to segment 3D medical images) and performed by the same rater. The inter‐rater intra‐class correlation (ICC) ranged from 0.918 to 0.998, suggesting excellent reproducibility of all brain regions. Moreover, because the manual corrections of the automated segmentations were completed by two raters (VS and IP), it was also necessary to establish reliability between the raters. Inter‐rater reliability of the manual corrections applied to the automated segmentations was excellent, with ICC values ranging between 0.823 and 0.986. For the total gray and white matter volume, the ICC of the inter‐rater variability was 0.973 (*P* < 0.001). Similar results were obtained for the individual correlations of each brain region: midbrain (0.823, *P* < 0.001), subcortical region (0.923, *P* = 0.001), cerebellum (0.982, *P* < 0.001), lateral ventricles (0.986, *P* < 0.001), and CSF (0.857, *P* < 0.001) (for more details please see the Supplementary Information Table S1).

### *Clinical and Behavioral Procedures*

#### *Mother–infant interactions at 4–6 months*

Within 2 weeks of the MRI scan (and usually on the same day), mother–infant interactions were video‐recorded using a standard assessment protocol (Murray, Hipwell, Hooper, Stein, & Cooper, 1996b) of a 5‐min face‐to‐face play session. Mothers were asked to play with and talk to their infant as they normally would, but without the use of any toys or objects, and with the infant seated facing towards the mother. Maternal and infant behavioral dimensions were coded by two trained raters experienced with the Global Ratings Scale (GRS; Murray, Hipwell, Hooper, Stein, & Cooper, 1996b) and blind to infant risk group.

The GRS is a valid and reliable tool for assessing mother–infant interactions in the context of ASD risk status. The measure is sensitive to a range of interactions among low‐risk (Cohn, Matias, Tronick, Connell, & Lyons‐Ruth, 1986; Gunning et al., 2004) and high‐risk samples, including mothers with depression (Murray, Fiori‐Cowley, et al., 1996a; Murray, Hipwell, Hooper, Stein, & Cooper, 1996b), schizophrenia (Riordan, Appleby, & Faragher, 1999), and borderline personality disorder (Crandell, Patrick, & Hobson, 2003). The scale has also been recently used with mothers of infants at risk of ASD (Blasi et al., 2015). Others examining this population have chosen an alternate measure developed with basis on the GRS and modified to better suit participant age range and risk status (Wan et al., 2012); however, the behaviors that they coded for parental sensitivity did not differ from ours. Our dimensions were rated on a 5‐point scale (1–5), with higher scores indicating more positive behaviors (for example, increased levels of maternal sensitivity) and lower scores indicating less optimal interactions (for example, low levels of maternal sensitivity). In cases where there were discrepancies between raters, these were discussed, and final consensus ratings were obtained in collaboration with the scale developers. The dimensions comprised:

1. *Maternal Sensitivity*: Maternal response to the infant's communication cues, and the extent to which it is contingent and appropriate to the infant's needs and experiences.
2. *Maternal Remoteness*: Maternal withdrawal and disengagement from the infant, which is manifested verbally, psychologically, and/or physically. A very remote mother may, for instance, create physical distance between herself and her infant or appear quiet and unresponsive, uncertain of what to do or say. Moments of detached behavior are also common among remote mothers, who at times appear to be lost in their own thoughts.
3. *Infant Communication*: Infant's level of engagement and communication, including positive vocal and non‐vocal behavior directed towards the mother.
4. *Infant Fretfulness*: Infant's affective state, including positive and negative affectivity.

Inter‐rater ICCs on a randomly selected 20% (i.e., *n* = 10) of the interactions ranged from 0.813 to 0.940, indicating acceptable inter‐rater reliability across all dimensions (but please see the Supplementary Information Table S2 for more information). Behavioral measures of observed mother–infant interactions were available for *n* = 23 of the 26 infants in the low‐risk group and *n* = 21 of the 24 infants in the high‐risk group.

#### *Behavioral assessment at 36 months*

At 36 months of age, all infants in the high‐risk group were invited back for a follow‐up behavioral assessment; 23 of the 24 infants scanned returned. Assessments included the Autism Diagnostic Observation Schedule (ADOS‐2; Lord et al., 2012), the Autism Diagnostic Interview (ADI‐R; Lord, Rutter, & Le Couteur, 1994), and the Mullen Scales of Early Learning (MSEL; Mullen, 1995). The MSEL is a standard instrument for the evaluation of cognitive ability and development; it is administered directly with the child and yields a standardized Early Learning Composite score of overall intellectual ability (MSEL ELC; mean = 100, SD = 15). The ADOS‐2 is a standard and semi‐structured play‐based assessment, used to examine autism‐related behavioral characteristics. Most children were administered Module 2 (designed for children with consistent phrase speech); however, *n* = 3 were assessed using Module 1 (designed for nonverbal children or children with only single words). To facilitate comparisons of symptom severity across different modules and ages, total algorithm scores were converted into standardized calibrated severity scores, ranging from 1 to 10 (Hus, Gotham, & Lord, 2014). Observations from the ADOS‐2 were complemented with a parent‐report of the infant's behaviors, administered using the ADI‐R structured interview. The ADOS‐2 and ADI‐R were also both used to assess behavioral outcomes because each provides different and complementary perspectives on a child's behavior. The ADOS‐2 provides an objective assessment of the child in person but at only one time‐point, whereas the ADI‐R depends upon parental impressions and examples of behavior gathered over time (Leekam, Prior, & Uljarevic, 2011).

In common with other research groups studying infants at risk of ASD (Zwaigenbaum et al., 2007), a “best estimate clinical consensus” approach to diagnosis was taken. That is, outcome classifications were determined by a group of experienced clinical researchers (TC, GP, CC), who considered all the available information (i.e., the MSEL, ADOS‐2, ADI‐R, and any informal observations) and agreed on a consensus ASD outcome based on the DSM‐5 criteria (American Psychiatric Association, 2013).

### *Statistical Analysis*

Statistical analysis was performed using SPSS 21.0 (IBM Corp, Armonk, NY), with significance set at *P* < 0.05. Normality of data distribution was assessed visually using box‐plots and statistically using the Shapiro–Wilk Test. Group differences in infant characteristics and mother–infant interaction dimensions were tested using the Independent Samples *t*‐test, the Mann–Whitney *U*‐test, and the Chi‐Square test, as appropriate.

#### *Baseline differences in regional brain volumes*

Brain volumes were not normally distributed; hence, logarithmic transformations were performed on all volumes to adjust for skew and achieve normal distribution. Analysis of covariance (ANCOVA) on the log‐transformed data was used to examine cross‐sectional differences in regional brain volumes between the two risk groups (low‐risk vs. high‐risk). Infant age and intracranial volume were included as covariates, and sex was included as a (between subjects) fixed factor because males and females are known to have different developmental trajectories of brain growth (Lenroot et al., 2007).

#### *Regional brain volumes and outcome measures within the high‐risk group*

In (subcortical and cerebellar) regions where the high‐risk group had shown significant differences in brain volumes at 4–6 months, we examined the association of these early volumes with ASD outcome measures (assessed by the ADOS‐2 and ADI‐R) at 36 months. Given that the ADOS‐2 calibrated severity scores and the ADI‐R scores were not normally distributed, dimensional analyses were run using nonparametric (Spearman's rank) correlations to explore associations between regional brain volumes at 4–6 months and observable autistic behaviors at 36 months.

#### *Moderation by mother–infant interactions*

Moderation analyses tested the effect of mother–infant interactions on the association between risk group and infant brain volume, in regions with significant group differences at 4–6 months. We applied the PROCESS macro tool (Hayes, 2012) to test whether the interaction between risk group and each of the four behavioral dimensions (maternal sensitivity and remoteness; infant communication and fretfulness) predicted infant cerebellar and subcortical brain volumes. Thus, eight tests were conducted overall. Covariates were infant age, sex, and intracranial volume; and given the small sample size, both unadjusted and adjusted results were presented. PROCESS applies bias corrected bootstrapping intervals to probe the interaction term and make inferences about indirect effects, rather than relying on the normality assumption. The number of bootstrap samples used to determine 95% bias‐corrected bootstrap confidence intervals was 1000. For continuous moderators, PROCESS produces the conditional effects of the independent variable at the sample mean of the moderator, and at plus and minus one standard deviation from the mean. A Bonferroni correction for multiple comparisons was applied. Where a significant interaction term emerged, the association between behavior and brain volume was further examined within each risk group, using partial Pearson correlations controlling for intracranial volume.

#### *Post hoc analyses*

Following assessment at 36 months, four male infants in the high‐risk group received a diagnosis of ASD. Analyses were repeated with the four ASD cases removed, to determine if the results observed were evident in children without a diagnosis and therefore representative of the high‐risk group as a whole, or whether they were mainly driven by individuals with an ASD diagnosis.

## Results

### *Sample Characteristics*

The risk groups did not differ significantly in age (at birth and at MRI), body weight (at birth and at MRI), or sex (Table 1). Also, at 36 months, when the high‐risk group infants were stratified by outcome group (*n* = 4 ASD vs. *n* = 19 non‐ASD), participants did not differ significantly in age (*P* = 0.896) or MSEL (*P* = 0.060). The ASD group did however score higher than the non‐ASD group on both the ADOS‐2 and ADI‐R measures, but please see Table 2 for more details.

**Table 1:** Infant Characteristics by Risk Group

Infant characteristics	Low‐risk group (*n* = 26)	High‐risk group (*n* = 24)	Group difference statistic, *P*‐value
Age at MRI (months); mean (SD)	4.81 (0.69)	4.79 (0.72)	*t* = 0.08, *P* = 0.937
Sex (male); *n* (%)	12 (46.15)	11 (45.83)	*χ* ^2^ = 0.00, *P* = 0.982
Gestational age at birth (weeks); mean (SD)	39.65 (1.69)	39.60 (1.16)	*t* = 0.12, *P* = 0.906
Body weight at birth (kg); mean (SD)	3.34 (0.49)	3.54 (0.54)	*t* = −1.40, *P* = 0.167
Body weight at MRI (kg); mean (SD)	7.00 (0.95)	7.34 (0.87)	*t* = −1.32, *P* = 0.195

*Note*. MRI was acquired at the 4–6‐month timepoint. SD = standard deviation.

**Table 2:** Infant Clinical and Behavioral Measures Acquired at 36 months and Split by Outcome Group

Measure	High‐risk non‐ASD group (*n* = 19)		High‐risk ASD group (*n* = 4)		Group difference
	Mean	SD	Mean	SD	Statistic, *P*‐value
Age at outcome visit (months)	38.97	1.54	38.86	0.89	*t* = 0.13, *P* = 0.896
MSEL ELC† at outcome visit	107.89	22.66	81.00	34.07	*t* = 1.99, *P* = 0.060
ADOS‐2 at outcome visit					
Social affect CSS	2.58	1.81	6.00	3.56	*U* = 13.50, *P* = 0.044*
Restricted and repetitive CSS	3.53	2.57	7.25	1.71	*U* = 9.50, *P* = 0.016*
Total CSS	2.11	1.73	6.00	4.24	*U* = 16.00, *P* = 0.081
ADI‐R† at outcome visit					
Social	1.63	1.34	16.00	6.68	*U* = 0.00, *P* < 0.001***
Communication	2.37	2.97	13.50	3.70	*U* = 0.50, *P* < 0.001***
Restricted and repetitive behaviors	0.53	0.96	6.00	0.82	*U* = 0.00, *P* < 0.001***

*Note*. †MSEL and ADI‐R measures were only available for *n* = 3 of the 4 infants in the high‐risk ASD group. MSEL ELC = Mullen Scale of Early Learning Composite Standard Score; ADOS‐2 = Autism Diagnostic Observation Schedule—Second Edition; CSS = calibrated severity score; ADI‐R = Autism Diagnostic Interview—Revised; SD = standard deviation. *P ≤ 0.05; ***P ≤ 0.001.

### *Group Differences in Mother‐Infant Interaction Dimensions at 4–6 Months*

There were no significant group differences in observed mother–infant interactions at 4–6 months for any of the dimensions examined (Table 3).

**Table 3:** Maternal and Infant Interaction Dimensions Acquired at 4–6 Months and Split by Risk Group

Interaction dimensions	Low‐risk group (*n* = 23)		High‐risk group (*n* = 21)		Group difference	
	Mean	SD	Mean	SD	*t*	*P*‐value
**Maternal dimensions**						
Sensitivity	3.70	0.75	3.44	0.59	1.24	0.223
Remoteness	4.61	0.54	4.45	0.87	0.72	0.474
**Infant dimensions**						
Communication	3.36	0.83	3.28	0.87	0.89	0.745
Fretfulness	4.09	0.73	4.13	0.71	0.76	0.886

*Note*. Interaction dimensions are scored on a scale from 1–5; low scores indicate poor interactions (for example, lower levels of sensitivity, fewer communication attempts, and increased infant fretfulness). SD = standard deviation.

### *Group Differences in Total Brain Matter and Intracranial Volume at 4–6 Months*

At 4–6 months, there was no significant difference in total brain matter [*F*(1,45) = 0.36, *P* = 0.549] or intracranium volume (i.e., head size) [*F*(1,45) = 0.33, *P* = 0.569] between infants in the high‐risk and low‐risk groups (Table 4). However, there was a main effect of age [total brain matter: *F*(1,45) = 35.63, *P* < 0.001; intracranium: *F*(1,45) = 24.16, *P <* 0.001] and sex [total brain matter: *F*(1,45) = 11.30, *P* = 0.002; intracranium: *F*(1,45) = 12.01, *P* = 0.001] across the sample; older infants and males had larger volumes. There were no significant age and/or sex interactions.

**Table 4:** Infant Brain Volumes Acquired at 4–6 Months and Split by Risk Group

Brain volumes (cm^3^)	Low‐risk group (*n* = 26)		High‐risk group (*n* = 24)		Group difference	
	Mean	SD	Mean	SD	*F*	*P*‐value
Intracranium^a^	839.68	86.23	852.93	80.05	0.33	0.569
Total brain matter^a^	702.56	71.55	714.29	69.03	0.36	0.549
Total gray and white matter	584.47	61.47	590.02	59.30	0.29	0.593
Midbrain	13.35	1.89	13.17	1.57	0.84	0.364
Subcortical region	33.59	2.99	35.27	3.34	4.64	0.037*
Cerebellum	71.15	8.91	75.83	8.09	6.92	0.012*
Lateral ventricles	14.22	5.67	13.34	3.73	0.59	0.448
Cerebrospinal fluid	122.90	27.23	125.30	31.75	0.03	0.857

*Note*. In this analysis of covariance, infant age and intracranial volume were included as covariates, and sex was included as a fixed factor. SD = standard deviation. *P ≤ 0.05.

a In this analysis, infant age was included as a covariate, and sex was input as a fixed factor.

Excluding the four participants who received an ASD diagnosis at 36 months did not materially alter these results.

### *Group Differences in Regional Brain Volumes at 4–6 Months*

**Figure 2:**
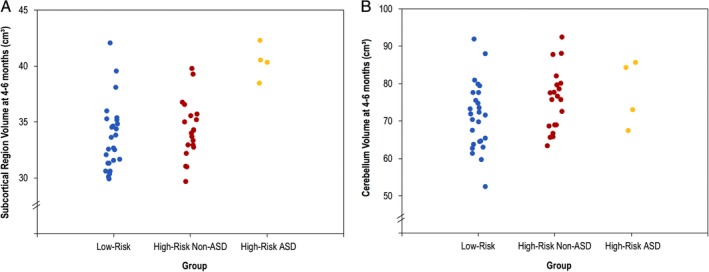
Individual data showing (A) subcortical and (B) cerebellum volumes (cm^3^) in 4–6‐month‐old infants with and without a familial risk of ASD; high‐risk infants have been further stratified according to whether they received an ASD diagnosis at 36 months.

At 4–6 months, infants in the high‐risk group had significantly larger cerebellum [*F*(1,44) = 6.92, *P* = 0.012] and subcortical volumes [*F*(1,44) = 4.64, *P* = 0.037] compared to the low‐risk group (Table 4, Fig. 2). There was a main effect of age only for the cerebellum; across the sample, older infants had larger volumes [*F*(1,44) = 16.63, *P <* 0.001]. In addition, there was a main effect of sex only for the subcortical region [*F*(1,44) = 4.31, *P* = 0.044]; males had larger volumes than females. There were no significant age and/or sex interactions.

When the four participants who received an ASD diagnosis were excluded, the cerebellar volume remained significantly larger in the high‐risk group [*F*(1,40) = 8.79, *P* = 0.005], with no significant difference in cerebellar volume between high‐risk individuals with and without an ASD diagnosis [*F*(1,18) = 1.07, *P* = 0.315]. In contrast, the subcortical region was no longer significantly enlarged in the high‐risk group [*F*(1,40) = 1.60, *P* = 0.213]; high‐risk children who received an ASD diagnosis had significantly larger subcortical volumes than those who did not receive a diagnosis [*F*(1,18) = 8.39, *P* = 0.010].

No other regional brain differences were identified between low‐risk and high‐risk infants.

### *Associations between Regional Brain Volumes at 4–6 Months and ASD Symptoms at 36 Months*

**Figure 3:**
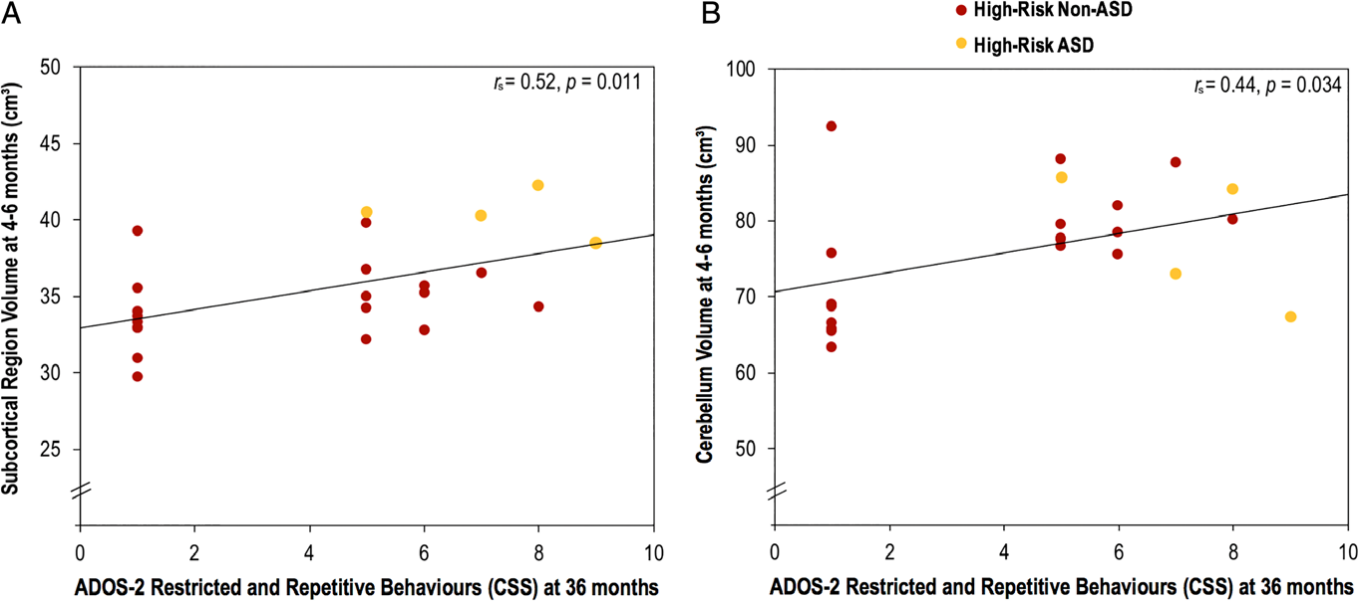
Scatter plots of the correlations between (A) subcortical and (B) cerebellum volumes of 4–6‐month‐old high‐risk infants and their restricted and repetitive behaviors at 36 months. The linear trendline indicates the correlation for all high‐risk infants, but those who received an ASD diagnosis at 36 months are highlighted in yellow. ADOS‐2 = Autism Diagnostic Observation Schedule—Second Edition; CSS = calibrated severity scores; *r*_*s*_ = Spearman's rank correlation coefficient.

Within the high‐risk group, there was a significant positive correlation between the volume of the subcortical region at 4–6 months and restricted and repetitive autistic behaviors at 36 months (ADOS‐2: *r*_s_ = 0.52, *P* = 0.011; ADI‐R: *r*_s_ = 0.72, *P* < 0.001) (Table 5, Fig. 3A). In addition, there was a trend level significance in social affect (ADOS‐2: *r*_s_ = 0.38, *P* = 0.071) and total autistic symptoms (ADOS‐2: *r*_s_ = 0.40, *P* = 0.061) within the high‐risk group, which disappeared when the four children who received an ASD diagnosis at 36 months were excluded from the analyses (Table 5). Moreover, although the association between subcortical volume at 4–6 months and restricted and repetitive behaviors at 36 months also disappeared when assessed using the ADOS‐2, it remained significant using the ADI‐R (*r*_s_ = 0.51, *P* = 0.026).

**Table 5:** Correlations Between Regional Brain Volumes at 4–6 Months and ASD Behaviors at 36 Months

Brain region	Behavioral Measure	High‐risk group (*n* = 23)		High‐risk non‐ASD group (*n* = 19)	
		*r*_s_	*P*‐value	*r*_s_	*P*‐value
Subcortical region	ADOS‐2 social affect CSS	0.38	0.071	0.19	0.447
	ADOS‐2 restricted and repetitive CSS	0.52	0.011*	0.36	0.132
	ADOS‐2 total CSS	0.40	0.061	0.26	0.275
	ADI‐R† social	0.54	0.007**	0.24	0.317
	ADI‐R† communication	0.49	0.018*	0.15	0.552
	ADI‐R† restricted and repetitive behaviors	0.72	0.001***	0.51	0.026*
Cerebellum	ADOS‐2 social affect CSS	0.35	0.104	0.51	0.027*
	ADOS‐2 restricted and repetitive CSS	0.44	0.034*	0.64	0.003**
	ADOS‐2 total CSS	0.32	0.143	0.55	0.014*
	ADI‐R† social	0.23	0.293	0.29	0.224
	ADI‐R† communication	0.21	0.343	0.19	0.439
	ADI‐R† restricted and repetitive behaviors	0.27	0.205	0.39	0.099

*Note*. †ADI‐R measures were only available for *n* = 3 of the 4 infants in the high‐risk ASD group. ADOS‐2 = Autism Diagnostic Observation Schedule—Second Edition; CSS = calibrated severity score; ADI‐R = Autism Diagnostic Interview—Revised; *r*_s_ = Spearman's rank correlation. *P ≤ 0.05; **P ≤ 0.01; ***P ≤ 0.001.

Among high‐risk infants, there was also a significant correlation between cerebellar volume at 4–6 months and restricted and repetitive behaviors at 36 months, as measured by the ADOS‐2 (*r*_s_ = 0.44, *P* = 0.034), but not the ADI‐R (*r*_s_ = 0.27, *P* = 0.205) (Table 5, Fig. 3B). The correlation between cerebellar volume and social affect was neither significant nor the association with total autistic symptoms. However, when the four high‐risk children who received an ASD diagnosis at 36 months were excluded from the analyses, correlations between cerebellar volume at 4–6 months and ADOS‐2 scores at 36 months became significant across all behavioral domains but remained nonsignificant for restricted and repetitive behaviors measured by the ADI‐R (please see Table 5).

### *Moderation of Regional Brain Volumes by Mother–Infant Interactions at 4–6 Months*

Maternal sensitivity moderated the association between infant risk group and volume of the subcortical region, suggesting an interaction between risk group and maternal sensitivity [risk group × maternal sensitivity: unadjusted (*β* = −0.05, *P* = 0.016, 95% CI = −0.08, −0.01)] (please see Supplementary information Fig. S1). Specifically, high‐risk infants whose mothers had lower levels of sensitivity tended to have the largest subcortical volumes at 4–6 months (high‐risk unadjusted: *r* = −0.45, *P* = 0.045 and adjusted: *r* = −0.45, *P* = 0.052; low‐risk unadjusted: *r* = 0.29, *P* = 0.187 and adjusted: *r* = 0.25, *P* = 0.271). The group difference between these correlation coefficients was significant (unadjusted: *z* = 2.37, *P* = 0.018; adjusted: *z* = 2.24, *P* = 0.025). However, the interaction term did not survive Bonferroni correction for multiple comparisons (*P* = 0.006) and was no longer significant when adjusting for infant age, sex, and intracranial volume [risk group × maternal sensitivity: adjusted (*β* = −0.03, *P* = 0.059, CI = −0.06, 0.00)].

Finally, when the four children who received an ASD diagnosis at 36 months were excluded from the analyses, the interaction between risk group and maternal sensitivity at 4–6 months was not significant despite a moderate effect size [risk group × maternal sensitivity: unadjusted (*β* = −0.03, *P* = 0.080, CI = −0.07, 0.00); adjusted (*β* = −0.03, *P* = 0.122, CI = −0.06, 0.01)].

Other maternal or infant behavior moderated neither the association between risk group and infant subcortical volume nor differences in cerebellar volumes.

## Discussion

In this study, we compared brain volumes between young infants at high‐risk and low‐risk of ASD and investigated whether early brain differences were associated with subsequent childhood emergence of autistic behaviors. At 4–6 months, there were no significant differences between high‐risk and low‐risk groups in head size (i.e., intracranial volume), total brain matter volume, or CSF volume. However, high‐risk infants had larger cerebellum and subcortical volumes at 4–6‐months. These early differences were also associated with the extent of autistic behaviors, especially repetitive behaviors in the high‐risk group at 36 months.

### *Cerebellum Findings*

Cerebellar abnormalities are among the most frequently reported findings in ASD literature [for reviews, see (Becker & Stoodley, 2013; Fatemi et al., 2012; Rogers et al., 2013)]. Most prior investigations, however, have been carried out in adolescents and adults with ASD and mainly report hypoplasia (usually vermal) rather than overgrowth (Courchesne, Yeung‐Courchesne, Press, Hesselink, & Jernigan, 1988; Levitt et al., 1999; McAlonan et al., 2002; Rojas et al., 2006). In contrast, studies of younger children with ASD have found enlargement of the cerebellum. For example, in studies of 2–5‐year‐olds, children with ASD were reported to have significantly larger total cerebellum (Sparks et al., 2002) and cerebellar white matter volume (Courchesne et al., 2001), as compared to typically developing controls. As predicted, our results extend these studies in children to younger ages and indicate that a familial risk of ASD may alter cerebellar development by as early as 4–6 months of age. Although the underlying cause(s) of this early enlargement (and subsequent lower size) remain to be established, it most likely reflects an abnormal regulation of growth (Courchesne et al., 2001; Sparks et al., 2002). Some have suggested that this may include an initial excess of neurogenesis/synaptogenesis, enlarged glia, premature dendritic/axonal growth, and incomplete synaptic pruning [for reviews, see (Bauman & Kemper, 2005; Palmen, van Engeland, Hof, & Schmitz, 2004)], followed by compensatory apoptotic and/or excitotoxic processes (Courchesne et al., 2001).

Across the high‐risk group, there was a significant association between cerebellum volume at 4–6 months and subsequent severity of restricted and repetitive behaviors as measured using the ADOS‐2 (but not the ADI‐R) at 36 months. The repetitive behaviors symptoms domain in young children has two main components, repetitive sensorimotor behaviors and ‘insistence on sameness’ (Leekam et al., 2011). It is known that observational measures of the repetitive symptoms domain may focus on different behaviors and yield distinct results from parental interview (Leekam et al., 2011), which is why we adopted a pragmatic approach and reported findings using both the ADI‐R and ADOS‐2.

Moreover, when high‐risk infants who received an ASD diagnosis were excluded from the outcome correlation analyses, the associations among cerebellum volume, repetitive behaviors, and other autistic symptoms became significant (with the exception of restricted and repetitive behaviors as measured on the ADI‐R). If the cerebellum is indeed an early marker for ASD, one could argue that the removal of the four ASD children should not have affected these results. Although removing these ASD individuals from the analyses reduced the sample size and power to identify significant differences, there may be other biological features in the diagnosed children beyond those defined here, which might have influenced the results. Another possibility is that the aberrant development of the cerebellum may be associated with a vulnerability to neurodevelopmental features in general, which is not necessarily specific to an ASD diagnosis. Indeed, cerebellar abnormalities have been identified in a range of developmental disorders, including ASD, ADHD, and developmental dyslexia (Stoodley, 2016).

In relation to the significant association we found between cerebellar volumes at 4–6 months and repetitive behaviors at 36 months, others have reported similar findings in both human and animal studies (D'Mello, Crocetti, Mostofsky, & Stoodley, 2015; Pierce & Courchesne, 2001; Rojas et al., 2006; Tsai et al., 2012; Wolff et al., 2017). In studies examining older individuals with ASD, smaller (D'Mello et al., 2015; Pierce & Courchesne, 2001; Rojas et al., 2006) and larger (D'Mello et al., 2015) cerebellar volumes have been linked to restricted and repetitive behaviors. For example, although restricted and repetitive behaviors have been associated with reduced gray matter in right Crus I/II and right lobules I–V, higher symptoms scores were also found to be associated with larger vermis VII and VIII (D'Mello et al., 2015). More recently, the structural properties of cerebellar white matter pathways in 6‐month‐old infants at high‐risk of ASD were associated with these behaviors and contributed to an ASD diagnosis at 2 years of age. Our findings align with these and suggest that the structure and volume of the cerebellum may be linked to restricted and repetitive behaviors among high‐risk infants from a very early age (Lord et al., 2006; Richler, Bishop, Kleinke, & Lord, 2007); however, some argue that these behaviors are not unique to children with ASD. Indeed, these behaviors also manifest in youngsters with other developmental disorders such as intellectual and language disabilities (Evans et al., 1997).

Finally, a relationship between cerebellum and social affect—which we only identified among high‐risk individuals without an ASD diagnosis at 36 months—has previously been reported in older cohorts with an ASD diagnosis but not in infants at high‐risk of ASD. The reason why we identified this pattern of results only once we excluded the four ASD infants is unclear. The diagnosis of ASD implies symptoms across multiple domains of functioning. By removing the children with an ASD diagnosis from this analysis, our sample characteristics shifted. Those remaining may have had sub‐clinical ASD traits in the social domain but not accompanied by sufficient other domain symptoms to warrant a diagnosis. This result does however require replication and further exploration in a larger sample.

In contrast to the association that we identified, studies of older individuals with ASD found that smaller gray matter volumes of cerebellum right lobule VI/Crus I and right lobule VIII were associated with more severe ratings on social communication scores (D'Mello et al., 2015; Rojas et al., 2006). Our findings were in the opposite direction, but we measured the entire cerebellum. Thus, we cannot say which subregion(s) of the cerebellum might be driving our results, and further studies will be needed to map the trajectory of cerebellar development and the emergence or change in autistic behaviors in later childhood.

### *Subcortical Findings*

We report that the subcortical region was significantly larger in 4–6‐month‐old infants at high‐risk of ASD compared to low‐risk peers. We also found that regardless of diagnostic classification, the size of the subcortical region in high‐risk infants at 4–6 months was correlated with repetitive behaviors at 36 months, as measured by the ADI‐R.

Although there are no published studies examining subcortical volume in infants at high‐risk of ASD, there are many reports that older individuals diagnosed with ASD have significant differences in the anatomy of subcortical structures. For example, the caudate nucleus has been reported to be larger in children, adolescents, and adults (Estes et al., 2011; Haznedar et al., 2006; Herbert et al., 2003; Hollander et al., 2005; Langen et al., 2007; Rojas et al., 2006; Sears et al., 1999), as well as to undergo an increased growth rate that is disproportional to overall brain growth (Langen et al., 2014). Thus, caudate overgrowth has been proposed to be a core abnormality of ASD (Stanfield et al., 2008). However, as described in a longitudinal study, authors did not identify caudate abnormalities in individuals with ASD but did report increased rates of putamen growth among ASD adolescents (Hua et al., 2013). Indeed, regions such as the globus pallidus and putamen have also been reported to be enlarged in individuals with ASD (Estes et al., 2011; Herbert et al., 2003; Hollander et al., 2005; Turner, Greenspan, & van Erp, 2016), although some of these findings may be confounded by differences in total brain volume (Estes et al., 2011; Herbert et al., 2003). Results from studies of the thalamus in ASD are less clear‐cut—with reports of no difference (Haznedar et al., 2006), larger volumes (Herbert et al., 2003), and smaller volumes (Hardan et al., 2006; Tsatsanis et al., 2003).

Subcortical volume differences have also been linked to ASD symptoms in older cohorts, although the direction of the relationship varies. For example, larger volume of the caudate in adolescents and adults has been reported to be both positively (Hollander et al., 2005; Rojas et al., 2006) and negatively (Sears et al., 1999) correlated with severity of repetitive and stereotyped behaviors and positively correlated with social and communication scores (Rojas et al., 2006). Similarly, studies of younger individuals also report contrasting results. Some have found that more restricted and repetitive behaviors are linked to smaller volumes of several subcortical regions (including the left thalamus, right globus pallidus, putamen, and striatum) in 3–4‐year‐olds (Estes et al., 2011). However, others report that faster striatal growth in preschool‐aged individuals with ASD is associated with more repetitive behaviors (Langen et al., 2014), and that 3‐6‐year‐olds with idiopathic autism show significant correlations between larger caudate volumes and more compulsive ritualistic behaviors (Wolff, Hazlett, Lightbody, Reiss, & Piven, 2013). Therefore, regardless of the direction of the association, work to date implicates abnormalities of the subcortical region with ASD symptoms, especially repetitive behaviors. Our study corroborates this, given that across the high‐risk group we identified an association between subcortical volume at 4–6 months and repetitive behaviors, as measured using the ADOS‐2 and ADI‐R at 36 months. However, when we removed the four ASD cases from the analyses, the result only held true when assessing repetitive behaviors using the ADI‐R. Although this was based on a smaller sample, which reduced the power to identify significant differences, an alternative explanation for why the association did not hold true across both measures may be because the ADOS‐2 and ADI‐R can yield different results. Although the ADOS‐2 provides an objective assessment of the child at a single time‐point, the ADI‐R provides parental report of behaviors over a more prolonged period of time (Leekam et al., 2011). Despite this, we did note that the removal of the four ASD children strengthened the cerebellum brain–behavior associations but weakened the subcortical brain–behavior correlations. Potentially what this suggests is that subcortical correlations are more linked to an ASD diagnosis, whereas cerebellar correlations are more generally associated with ASD traits.

### *Influence of the Parent–Child Environment*

Finally, we also found a preliminary indication that the association between risk group and infant subcortical volume is moderated by maternal sensitivity during observed mother–infant interactions at 4–6 months. Specifically, high maternal sensitivity was associated with less enlargement of this region in the high‐risk group. This did not survive adjustment for covariates or multiple comparisons, and when the four children who received an ASD diagnosis were excluded, the result was no longer significant. Although the smaller sample size reduced power, it may suggest that the original observation was driven by the infants who would go on to receive an ASD diagnosis. One possibility is that these children were already expressing some symptoms at 4–6 months, and that this was picked up during the parent–child interaction. Another possibility is that the covariates (age, sex, and intracranial volume) or other factors could have contributed to the moderation effect identified.

Future larger‐scale studies will be needed to provide a more robust assessment of how the two‐way parent–child environment links to brain biology and vice versa.

### *Relevant Negative Findings*

In contrast to recent studies of infants with a familial risk of ASD (Shen et al., 2013, 2017), we did not identify greater extracerebral CSF volume in the high‐risk group. There are several reasons that could explain the divergent results. First, this study focussed on infants aged 4–6 months, whereas other studies have examined infants aged 6–9 months and older; it could be that enlarged extracerebral CSF volume is only present in individuals older than 6 months. Second, in both studies, Shen et al., (2013, 2017) drew a horizontal slice through the anterior commissure to define a ventral boundary for the region of the extracerebral CSF. The present study did not take this approach, and therefore it is conceivable that differences in the anatomical delineation of the region may explain divergent results. Finally, although the 2013 study by Shen et al., had a similar sample size to that of the present study (high‐risk: *n* = 33 vs. *n* = 24; low‐risk: *n* = 22 vs. *n* = 26), the more recent 2017 study replicated the prior findings with a much larger sample, and therefore, the possibility of a false negative error in our study cannot be excluded.

### *Study Limitations*

This study has several limitations. First, given that our sample was of a modest size, we focused on a comparison of high‐ and low‐risk infants at 4–6 months, and explored the association between regional brain volumes and autistic symptoms across the entire high‐risk group. Our design focused on the relationship between brain and behavior and was not powered to compare children with and without a diagnosis of ASD. A further comparison of high‐risk infants with and without a diagnosis of ASD at 36 months in larger cohorts is currently underway as part of “EU‐AIMS”—a multicenter study seeking to identify early biomarkers of ASD (Murphy & Spooren, 2012). Second, because we were not funded to collect the same behavioral data from both risk groups, outcome data was only collected for high‐risk infants and not low‐risk infants, which restricted the interpretation of our findings. Third, but in line with what has been reported by others (Hazlett et al., 2012), the differentiation of gray and white matter was not possible due to ongoing myelination at this age. Fourth, due to the low resolution of our images, which was a pragmatic decision to include as many participants as possible, we could not separate the cerebellum or subcortical region into smaller components. Fifth, given the small sample size of this study and our a priori hypothesis, no correction for multiple comparisons was conducted when examining mean volumetric differences between risk group or when conducting correlation analysis between brain and behavior among the high‐risk group. The possibility of a false positive (type I) error cannot therefore be excluded. Sixth, our measures of parenting interactions were limited to cross‐sectional observations, which might not capture a sustained trait but rather a temporary state of interaction. We also acknowledge that the measures of mother–infant interactions were conducted in an unnatural research setting. This may have exacerbated maternal remoteness and infant fretfulness, and it is also possible that it affected groups unequally. Finally, our parenting interactions were also limited to observations with only the mothers, and further research would benefit from the inclusion of repeated measures of longitudinal observations with both mothers and fathers.

## Conclusions

To our knowledge, this is the first MRI study to report that subcortical and cerebellar brain volume differences in infants at high‐risk of ASD are present before 6 months of age and correlate with subsequent ASD trait severity. Further investigation is warranted to establish the utility of the cerebellum and subcortical region as potential “risk” markers for this domain of symptoms.

## Funding Disclosure

This paper represents independent research part funded by the National Institute for Health Research (NIHR) Biomedical Research Centre at South London and Maudsley NHS Foundation Trust and King's College London (Medical Research Council grant no. G0400061 to DGMM). The views expressed are those of the authors and not those of the NHS, the NIHR, or the Department of Health. This work was also supported by a Medical Research Council (MRC) Program Grant (no. G0701484 to MHJ), the Simons Foundation (grant no. SFARI201287 to MHJ), the BASIS funding consortium led by Autistica (www.basisnetwork.org), the developing human connectome project (dHCP) under the European Union's Seventh Framework Programme (grant FP7/2007‐2013; ERC grant agreement no. 319456), EU‐AIMS, and AIMS‐2 TRIALS. EU‐AIMS receives support from the Innovative Medicines Initiative (IMI) Joint Undertaking (JU) under grant agreement no. 115300, the resources of which are composed of financial contributions from the European Union's Seventh Framework Programme (grant FP7/2007‐2013), from the European Federation of Pharmaceutical Industries and Associations (EFPIA) companies' in‐kind contributions, and from Autism Speaks. AIMS‐2 TRIALS received funding from the IMI 2 JU under grant agreement no. 777394, with support from the European Union's Horizon 2020 research and innovation programme, EFPIA, AUTISM SPEAKS, Autistica, and SFARI. DGMM, GMM, TC, SCRW, and MJ are all part of the EU‐AIMS consortium. DGMM and GMM also receive support from the Sackler Centre for Translational Neurodevelopment at King's College London. MHJ, TC, AB, and SLF are additionally supported by the UK Medical Research Council. SCRW is additionally funded by the Wellcome Trust.

## Ethical Standards

This study received ethics approval from the UK National Research Ethics Service (REC 08/H0718/76 and 06/MRE02/73). The authors assert that all procedures contributing to this work comply with the ethical standards of the relevant national and institutional committees on human experimentation and with the Helsinki Declaration of 1975, as revised in 2008.

## Conflict of Interest

No conflicts declared.

## Supporting information


**Supplementary Figure 1:** A representation of the interaction between maternal sensitivity and risk group (i.e. low‐risk vs. high‐risk) on infant subcortical volume (cm^3^) at 4–6 months. Please note that this interaction did not survive correction for covariates or multiple comparisons, and when the 4 children who received an ASD diagnosis were excluded, the result was no longer significant. The high‐risk infants who received a diagnosis of ASD at 36 months are highlighted in yellow. Linear trendlines have been fitted to the risk groups (red: high‐risk; blue: low‐risk) – not the outcome groups – because the association between maternal sensitivity and subcortical volume was examined within each risk group individually.

**Supplementary Table S1:** Inter‐rater reliability of the manual corrections applied to the automated brain volume segmentations
**Supplementary Table S2:** Inter‐rater reliability of the behavioral coding for the mother‐infant interaction dimensions
