# Supplementary figures and images for: Familial risk of autism alters subcortical and cerebellar brain anatomy in infants and predicts the emergence of repetitive behaviors in early childhood
Source: Autism Res. 2019 Feb 22;12(4):614–27. doi: 10.1002/aur.2083 (PMC6519039; doi:10.1002/aur.2083)

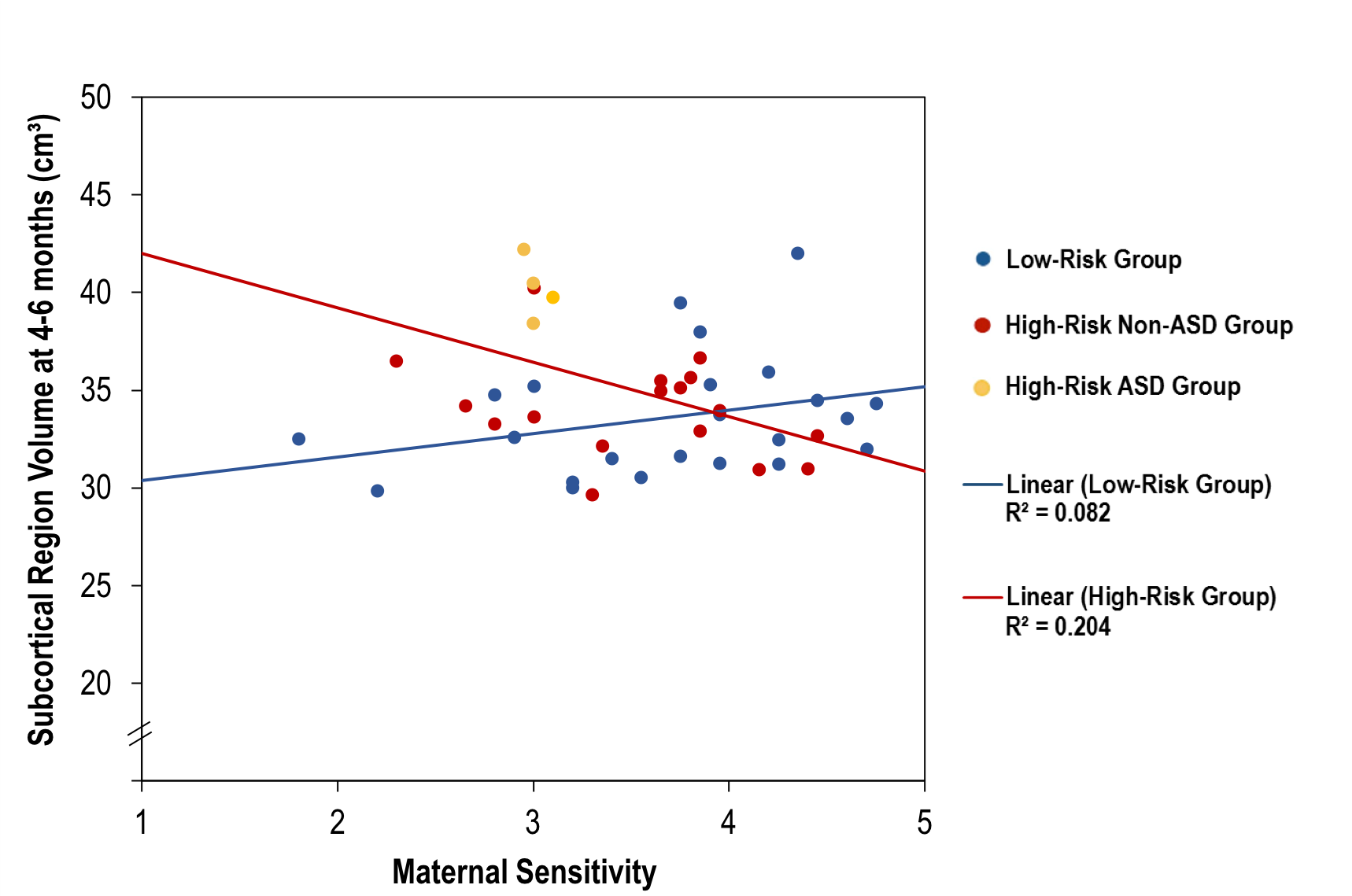

Supplement: Supplementary file 1 — Supplementary Figure 1: A representation of the interaction between maternal sensitivity and risk group (i.e. low‐risk vs. high‐risk) on infant subcortical volume (cm3) at 4–6 months. Please note that this interaction did not survive correction for covariates or multiple comparisons, and when the 4 children who received an ASD diagnosis were excluded, the result was no longer significant. The high‐risk infants who received a diagnosis of ASD at 36 months are highlighted in yellow. Linear trendlines have been fitted to the risk groups (red: high‐risk; blue: low‐risk) – not the outcome groups – because the association between maternal sensitivity and subcortical volume was examined within each risk group individually. [file AUR-12-614-s001.png]
