# Supplementary material for: Familial risk of autism alters subcortical and cerebellar brain anatomy in infants and predicts the emergence of repetitive behaviors in early childhood
Source: Autism Res. 2019 Feb 22;12(4):614–27. doi: 10.1002/aur.2083 (PMC6519039; doi:10.1002/aur.2083)
Supplement: Supplementary file 2 — Supplementary Table S1: Inter‐rater reliability of the manual corrections applied to the automated brain volume segmentations Supplementary Table S2: Inter‐rater reliability of the behavioral coding for the mother‐infant interaction dimensions [file AUR-12-614-s002.docx]

**Supplementary Material**

**Supplementary Table S1: Inter-rater reliability of the manual corrections applied to the automated brain volume segmentations**

Note: ICC = inter-rater intra-class correlation

| **Total Grey & White Matter Volume (cm^3^)** | | **Midbrain Volume (cm^3^)** | | **Subcortical Region Volume (cm^3^)** | | **Cerebellum Volume (cm^3^)** | | **Lateral Ventricular Volume (cm^3^)** | | **CSF Volume (cm^3^)** | |
| --- | --- | --- | --- | --- | --- | --- | --- | --- | --- | --- | --- |
| **Rater 1** | **Rater 2** | **Rater 1** | **Rater 2** | **Rater 1** | **Rater 2** | **Rater 1** | **Rater 2** | **Rater 1** | **Rater 2** | **Rater 1** | **Rater 2** |
| 704.95 | 705.335 | 12.211 | 10.025 | 34.045 | 32.247 | 82.71 | 82.846 | 28.377 | 27.982 | 142.987 | 138.514 |
| 626.992 | 626.677 | 10.343 | 8.964 | 31.396 | 28.937 | 87.877 | 87.832 | 28.786 | 29.287 | 140.902 | 140.021 |
| 565.181 | 562.076 | 11.099 | 11.123 | 35.353 | 34.276 | 63.908 | 64.436 | 13.054 | 13.027 | 92.886 | 91.872 |
| 645.666 | 624.26 | 9.859 | 8.961 | 31.791 | 33.585 | 79.103 | 71.516 | 15.7 | 19.128 | 109.434 | 142.207 |
| 529.665 | 529.665 | 10.921 | 9.548 | 31.791 | 31.951 | 54.289 | 54.289 | 14.486 | 14.486 | 113.424 | 113.424 |
| 593.923 | 572.819 | 10.375 | 9.874 | 28.925 | 30.127 | 79.349 | 79.907 | 13.256 | 13.709 | 103.594 | 133.074 |
| 564.549 | 531.044 | 7.59 | 7.175 | 29.709 | 29.005 | 62.324 | 62.739 | 13.196 | 14.914 | 150.203 | 182.557 |
| 648.442 | 615.531 | 10.992 | 9.397 | 31.266 | 31.98 | 75.44 | 74.66 | 15.981 | 17.817 | 140.617 | 170.239 |
| 583.743 | 586.353 | 9.331 | 8.892 | 32.336 | 32.645 | 78.136 | 76.374 | 14.759 | 14.825 | 96.695 | 96.33 |
| 539.839 | 522.081 | 12.007 | 11.805 | 36.542 | 37.714 | 67.497 | 63.62 | 9.373 | 11.247 | 153.267 | 164.639 |
| **ICC = 0.973, *p* < 0.001** | | **ICC = 0.823, *p* < 0.001** | | **ICC = 0.923, *p* < 0.001** | | **ICC = 0.982, *p* < 0.001** | | **ICC = 0.986, *p* < 0.001** | | **ICC = 0.857, *p* < 0.001** | |

**Supplementary Table S2: Inter-rater reliability of the behavioural coding for the mother-infant interaction dimensions**

| **Maternal Sensitivity** | | **Maternal Remoteness** | | **Infant Communication** | | **Infant Fretfulness** | |
| --- | --- | --- | --- | --- | --- | --- | --- |
| **Rater 1** | **Rater 2** | **Rater 1** | **Rater 2** | **Rater 1** | **Rater 2** | **Rater 1** | **Rater 2** |
| 2.85 | 3.00 | 4.88 | 4.50 | 4.17 | 3.33 | 3.50 | 4.00 |
| 3.95 | 4.15 | 5.00 | 4.50 | 1.50 | 2.33 | 3.50 | 3.00 |
| 3.85 | 3.30 | 5.00 | 5.00 | 3.33 | 3.00 | 3.00 | 3.25 |
| 3.95 | 4.00 | 3.00 | 3.00 | 2.50 | 2.00 | 4.00 | 3.00 |
| 2.20 | 2.80 | 4.50 | 4.00 | 3.00 | 3.17 | 5.00 | 4.50 |
| 3.00 | 3.60 | 5.00 | 4.50 | 2.50 | 3.00 | 2.25 | 2.00 |
| 1.70 | 2.40 | 5.00 | 4.50 | 3.00 | 3.67 | 2.75 | 3.00 |
| 3.75 | 3.80 | 4.00 | 4.00 | 3.83 | 3.67 | 4.50 | 4.50 |
| 3.65 | 3.60 | 4.88 | 4.00 | 4.50 | 4.00 | 5.00 | 5.00 |
| 3.80 | 4.20 | 5.00 | 5.00 | 4.00 | 3.00 | 4.00 | 4.00 |
| **ICC = 0.908, *p* < 0.001** | | **ICC = 0.876 , *p* < 0.001** | | **ICC = 0.813, *p* = 0.012** | | **ICC = 0.940, *p* < 0.001** | |

Note: ICC = inter-rater intra-class correlation
